# MUAC-for-age more useful than absolute MUAC for nutritional surveillance in Somalia: results from nineteen cross-sectional surveys (2007–2016)

**DOI:** 10.1186/s40795-018-0213-3

**Published:** 2018-02-21

**Authors:** Estefania Custodio, Rocio Martin-Cañavate, Federica Di Marcantonio, Daniel Molla, Yusuf Abukar, Francois Kayitakire

**Affiliations:** 1 European Commission, Joint Research Centre, Via E. Fermi, 2749, 21027, Ispra, Varese, Italy; 2 Food Security and Nutrition Analysis Unit - Somalia, United Nations Food and Agriculture Organization, P.O. Box 30470-00100, Ngecha Road, Nairobi, Kenya

**Keywords:** Acute malnutrition, Weight-for-height, MUAC, MUAC-for-age, Severe acute malnutrition, Children, Somalia, Africa

## Abstract

**Background:**

Somalia is affected by a civil war and a protracted humanitarian crisis for more than two decades. The international community has put in place nutrition surveillance systems to monitor the situation and inform decisions. However, the indicators commonly used to identify acute malnutrition, weight-for-height Z-score (WHZ) and mid upper arm circumference (MUAC), do not always converge in their estimations of acute malnutrition, creating challenges for decision making. Furthermore, the divergences are not consistent across livelihood populations within the country. We explored the MUAC-for-age Z-score (MUACAZ) as an alternative indicator in Somalia to minimize the discrepancy.

**Methods:**

We analyzed data from nineteen cross-sectional surveys conducted in Somalia between 2007 and 2016. We compared the acute malnutrition prevalence estimates by each of the indicators and the degree of overlap in the individual diagnosis of acute malnutrition between the WHZ and the MUAC-based indicators. We performed multivariate regression analysis with sex, age and stunting as independent variables and acute malnutrition as the dependent outcome, defined by WHZ, MUAC or MUACAZ. We performed all the analysis in the population overall and in each of the livelihood populations separately.

**Results:**

A total 255,623 measurements of children 6–59 months of age were analyzed. The overall prevalence of global acute malnutrition by MUACAZ (15.8%) was similar to the one obtained using WHZ (16%), whereas prevalence based on MUAC was much lower (7.8%). These patterns of divergence were sustained throughout the nineteen surveys and the livelihoods studied, with only few exceptions. However, the proportion of overlap in the individual diagnosis of children as acutely malnourished was low between WHZ and absolute MUAC diagnosis (18.1%) and also between WHZ and MUACAZ (28.3%). Results show that age, sex and stunting status of the child affected the likelihood of being diagnosed as acutely malnourished to varying degrees, depending on the indicator used.

**Conclusions:**

The MUAC-for-age (MUACZ) indicator yielded acute malnutrition prevalence estimates convergent with those obtained by WHZ indicator. However, the degree of overlap between these two indicators for individual diagnosis of acute malnutrition is low. Further studies of MUACAZ as an alternative indicator for nutrition surveillance are needed.

**Electronic supplementary material:**

The online version of this article (10.1186/s40795-018-0213-3) contains supplementary material, which is available to authorized users.

## Background

Somalia has been affected by a civil war since 1991, with the food security situation further aggravated by recurrent and prolonged droughts. Located in the Horn of Africa, the combination of conflict and natural disasters has eroded its livelihoods, caused structural food insecurity, population displacements and extreme poverty [1]. The country has experienced two famines in its recent history: 1992/1993 and 2011/2012 and it is currently one of the four countries (including South Sudan, Nigeria and Yemen) that are facing risk of famine in 2017. Within this context, the rates of acute malnutrition have persistently remained at ‘critically’ high levels over the last two decades [2]. As part of the efforts of the international community to improve the conditions in the country, the Somalia Food and Nutrition Security Analysis Unit (FSNAU) has been collecting data on anthropometric indicators throughout time to monitor the situation.

On-going surveillance of anthropometric indicators is crucial to detect the deterioration of the nutritional status of a population. It can provide information on trends, allowing for comparison overtime and against baseline years results, and it permits for geographical and contextual comparisons which can inform the prioritization of actions and the allocation of resources [3]. For this purpose, however, it is essential that the indicators used to monitor the situation yield comparable results.

In two recent reviews of nutrition surveillance systems in humanitarian settings and low income countries [3, 4], the advantages and challenges of the different data collection systems were discussed in terms of validity, reliability and institutional management, but little attention was given to the anthropometric measures being collected, which have been discussed elsewhere [5].

The recommended anthropometric indices and measures for acute malnutrition surveillance are weight-for-height/length Z-score (WHZ), absolute value of Mid-Upper Arm Circumference (MUAC) and MUAC-for-age Z-score (MUACAZ), with the cut-off points detailed in Box 1, being WHZ and MUAC the most widely used.

However, and although the absolute MUAC cut-off points were selected from statistical analysis to best match the WHZ cut-off points, so that the same prevalence of acute malnutrition would be found with each criterion [6], it has been shown that the prevalence obtained when using either indicator are not convergent and that the children identified for admission to programs with either indicator are not the same [7, 8].

For this reason, in nutrition surveillance systems aimed at identifying children with severe acute malnutrition (SAM) for admission in feeding programs, the World Health Organization (WHO) and United Nations International Children’s Emergency Funds (UNICEF) recommend the use of WHZ < − 3 and also the use of absolute MUAC below 115 mm, as the latter indicator presents operational advantages in terms of time and cost of data collection, and is also believed to identify the children at higher risk of death [9]. Although the discussion regarding which of these two indicators is more correlated with mortality is still on-going [7, 10], the use of absolute MUAC in combination with or in replacement of WHZ has been spread for program screening by field organizations, as a practical tool to screen vulnerable children in need for treatment [11].

However, if the aim of the surveillance is to assess the severity of the situation in the population and the targeting of humanitarian actions, WHO has recommended the use of WHZ or the use of MUAC adjusted by age and sex, that is the MUACAZ, but not the use of absolute MUAC [12]. The main reason is that WHZ and MUACAZ take into account the sex and the age or the height of the child, whereas absolute MUAC is strongly age- and sex- dependent, thus malnutrition estimates are conditioned to the age and sex structure of the study population when this latter indicator is used [13]. However, as MUACAZ requires accurate age data collection, which may be a challenge in certain humanitarian contexts, the preferred indicator has been the WHZ, and it is the only indicator of acute malnutrition for which there are prevalence thresholds to support the public health interpretation of results at global level [12].

Nonetheless, in recent times, the use of the MUAC measurement (or absolute MUAC) below 125 mm has been advocated as a proxy for global acute malnutrition for population estimates, grounded on the operational advantages of the indicator described before [5]. This is creating major confusion amongst policy and decision makers, as the discrepancies in the acute malnutrition estimates when using the WHZ index or the absolute MUAC measurement are not consistent across populations [11]. This is highly problematic in terms of geographical and temporal comparisons of the nutrition situation across populations, even at the region or sub-region level within a country.

The reasons for these discrepancies are not fully understood, but an important part of this variation must be linked to the age and sex dependency of the absolute MUAC measurement [8]. That is why MUACAZ was suggested as a more useful indicator of nutritional status for population estimates than absolute MUAC [14]. Moreover, the MUACAZ has been shown to be a sensible indicator to detect changes in the nutritional status of populations [15]. Therefore, and as it retains most of the operational advantages of the MUAC measurement, it may be an alternative indicator when the weight and height measurements are difficult to obtain. Nevertheless, little is known of how the acute malnutrition estimates using MUACAZ relate to the ones obtained using WHZ, as the MUACAZ indicator has been rarely used. Therefore, we propose to bring some light on this topic by exploring anthropometric data in a context of high acute malnutrition prevalence like Somalia.

For this purpose we compare the nutrition outcomes yielded by absolute MUAC and MUACAZ with those obtained using WHZ in different populations of Somalia over the 2007–2016 period. We analyse also the effect of factors, namely sex, age and stunting status, expected to contribute to the discrepancy observed between malnutrition rates derived from the three indicators.

## Methods

The data used for this study were obtained from 19 surveys undertaken by FSNAU and partners working in Somalia, in a biannual basis, in the 2007–2016 time period.

There are three main livelihoods in the country: the pastoralists; the agro-pastoralists; and the riverines, that are mainly agrarian [2]. Because of the conflict undergoing in the country, FSNAU also collects data on Internally Displaced Persons (IDPs) which represent a significant proportion of the Somali population. Surveys of IDP camps were included, and these were coded as a further livelihood category although it doesn’t constitute a livelihood sensu stricto.

A two-stage cluster sampling with probability proportional to size design was employed where sample sizes for the surveys (number of households and number of children) were calculated using Standardized Methodology for Survey in Relief and Transition (SMART) methods. Previous estimations of global acute malnutrition measured by WHZ and crude mortality rates for the surveyed areas were used for the calculations. An additional 10% was added to the sample size to allow for dropout or refusal to participate.

The data were cleaned by deleting the records of individual children with any of the following criteria: age < 6 months, age > 59 months, and age, sex, weight, height, oedema or MUAC not recorded. Cases of oedematous malnutrition were excluded from analysis because the WHZ indicator is not valid as proxy of acute malnutrition in such cases (*n* = 650, 0.25% of the total sample). Weight-for-height and MUAC-for-age Z-scores were calculated using WHO Anthro (version 3.2.2, January 2011) and macros using WHO 2006 growth standards. Extreme biologically implausible values were excluded based on WHO standards with recommended flag limits of WHZ and MUAC Z-scores − 5, + 5 [16].

We defined global acute malnutrition (GAM) and severe acute malnutrition (SAM) using the indexes and cut offs described in Table 1 below.

**Table 1:** Common anthropometric measurements and indices for children under 5 years of age

Index/measurement	Nutritional condition	Indicator	(Acronym)
Weight-for-height/length Z score	Global acute malnutrition	WHZ < − 2	(WHZ_-2)_
	Severe acute malnutrition	WHZ < − 3	(WHZ_-3)_
MUAC	Global acute malnutrition	MUAC < 125 mm	(MUAC_125)_
	Severe acute malnutrition	MUAC < 115 mm	(MUAC_115)_
MUAC-for-age Z score	Global acute malnutrition	MUACAZ <− 2	(MUACAZ_-2)_
	Severe acute malnutrition	MUACAZ <− 3	(MUACAZ_-3)_

We calculated the prevalence of GAM according to each of the three indicators in a sample pooling the data of the nineteen surveys, stratified by livelihood system, and then, for each of the surveys separately.

For comparing the diagnosis of acute malnutrition using WHZ and absolute MUAC, we defined *WHZ*_*− 2*_ *only* or *WHZ*_*− 3*_ *only* when the child had WHZ below − 2 or WHZ below − 3 but MUAC over 125 mm or 115 mm respectively, and *MUAC*_*125*_ *only* or *MUAC*_*115*_ *only* when MUAC was below 125 or below 115 but WHZ was equal or greater than − 2, or above − 3, respectively. When comparing WHZ and MUACAZ the *WHZ*_*− 2*_ *only* or the *WHZ*_*− 3*_ *only* refer to children identified as GAM or SAM by WHZ but not by MUACAZ, and the *MUACAZ*_*− 2*_ *only* and the *MUACAZ*_*− 3*_ *only* to children with MUACAZ below − 2 or − 3 but above these same values for WHZ.

We performed multivariate logistic regressions on GAM and SAM prevalence defined by WHZ, absolute MUAC and MUACAZ. The independent variables used were sex (male vs female), young age (age < 24 months vs. age ≥ 24 months), and stunting (height for age Z score < − 2 vs height for age Z score > − 2), as they are the hypothetical contributors to the discrepant malnutrition diagnosis between WHZ and absolute MUAC [8]. We tested the association of these independent variables with either WHZ_− 2_, MUAC_125_ or MUACAZ_− 2_ for GAM and WHZ_− 3_, MUAC_115_ or MUACAZ_− 3_ for SAM including all covariates (i.e. sex, young age and stunting) in the overall sample and then in each of the livelihood samples separately, to control for potential confounding and mediating effects.

Stata 14 (StataCorp, College Station, Texas, USA) was used for statistical analysis.

## Results

A total of 255,623 measurements of children aged 6 to 59 months from 19 surveys were examined. There was a nearly equal proportion of girls and boys (49% girls), and the mean age of the children was 31.6 (±0.03) months, with a distribution of 66% children with 24 months or older and 33% children between 6 months and 24 months of age. By livelihood system, 16.5%, 35.2% and 10.4% of children were from areas of agro-pastoral, pastoral and riverine livelihoods, respectively, while 38% lived in IDPs camps.

### Population estimates of acute malnutrition

In Table 2 we present the prevalence of acute malnutrition (GAM and SAM) as measured by any of the three indicators for the total population, and in each of the livelihoods in Somalia, using the pooled sample of the 2007–2016 surveys.

**Table 2:** Prevalence of acute malnutrition, defined by WHZ^a^, absolute MUAC^b^ and MUACAZ^c^ by four Somalian livelihoods

		WHZ				MUAC				MUACAZ			
	Total subjects	WHZ_−2_		WHZ_−3_		MUAC_125_		MUAC_115_		MUACAZ_−2_		MUACAZ_−3_	
Livelihood	*n*	%	95% CI	%	95% CI	%	95% CI	%	95% CI	%	95% CI	%	95% CI
Agro-pastoralist	42,132	16.3	(16.0–16.7)	3.9	(3.8–4.1)	9.6	(9.4–9.9)	1.8	(1.6–1.9)	19.3	(18.9–19.7)	4.2	(4.0–4.4)
Pastoralist	89,876	15.5	(15.2–15.7)	3.2	(3.1–3.4)	5.7	(5.5–5.8)	1.0	(0.9–1.1)	13.3	(13.1–13.6)	2.2	(2.1–2.3)
Riverine	26,603	16.0	(15.6–16.5)	3.9	(3.6–4.1)	8.9	(8.6–9.3)	1.7	(1.6–1.9)	18.2	(17.7–18.7)	3.5	(3.3–3.8)
IDPs	97,012	16.5	(16.3–16.7)	3.9	(3.8–4.0)	8.7	(8.5–8.9)	1.9	(1.8–2.0)	15.9	(15.7–16.1)	3.0	(2.9–3.1)
Total	255,623	16.1	(15.9–16.2)	3.7	(3.6–3.8)	7.8	(7.7–7.9)	1.5	(1.4–1.6)	15.8	(15.7–15.9)	3.0	(2.9–3.1)

^a^GAM by WHZ defined as WHZ < −2 from the reference population (WHZ_− 2_) and SAM by WHZ defined as WHZ < −3 compared to the reference population (WHZ_− 3_)

^b^GAM by MUAC defined as MUAC< 125 mm (MUAC_125_) and SAM by MUAC defined as MUAC< 115 mm (MUAC_115_)

^c^GAM by MUACAZ defined as MUACZ<− 2 from the reference population (MUAC_− 2_) and SAM by MUACAZ defined as MUACAZ<− 3 compared to the reference population (MUACAZ_− 3_)

The prevalence of global acute malnutrition in the total population was very close when calculated by WHZ_− 2_ and by MUACAZ_− 2_ (16% vs. 15.8%), and half of it when calculated by MUAC_125_ (7.8%). In pastoralists, the difference was even greater, as the prevalence of GAM by MUAC_125_ was less than half the GAM prevalence yielded by the other two indicators. The SAM estimates showed similar patterns to GAM (Table 2).

**Fig. 1:**
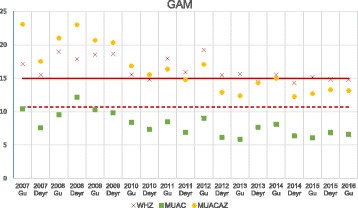
Prevalence of GAM by survey as measured by WHZ, absolute MUAC or MUACAZ in Somalia (2007–2016). The WHO emergency threshold for GAM by WHZ > 15% (red line) and the FSNAU emergency threshold for GAM by absolute MUAC > 10.7% (red dashed-line) are represented

The discrepancies between the estimations of GAM by each of the three indicators were sustained throughout the 19 surveys (Fig. 1). In all the surveys the prevalence of GAM was above 12% when using WHZ_− 2_ orMUACAZ_− 2_ and much lower when (below 10% in most of the surveys) using MUAC_125_.

### Acute malnutrition screening

**Fig. 2:**
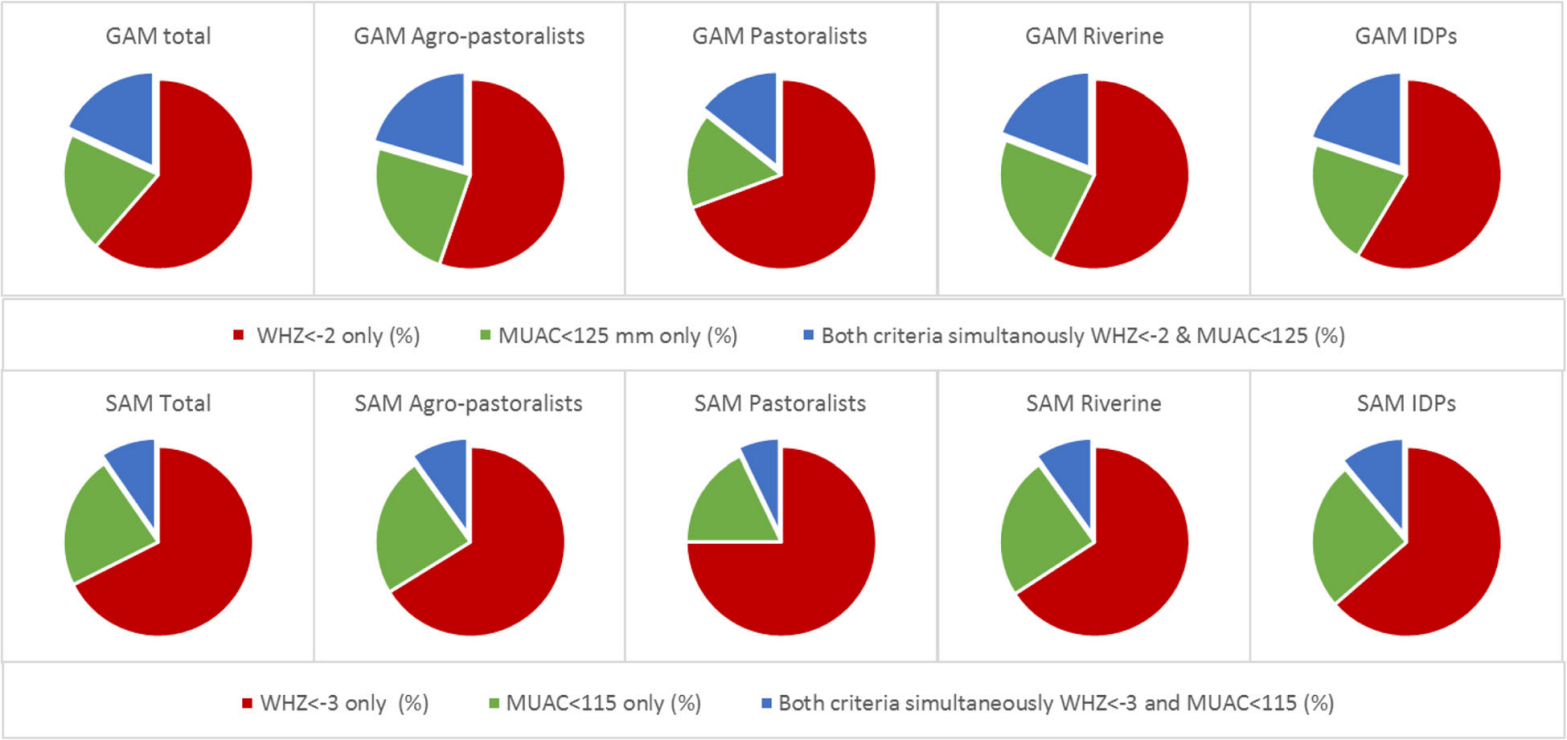
Comparison of acute malnutrition diagnosis by WHZ and/or absolute MUAC in four Somalian livelihoods. Proportion of acutely malnourished children that are diagnosed as such (GAM first row and SAM second row) either by WHZ alone (red), by absolute MUAC alone (green) or by both WHZ and absolute MUAC (green), for the total population and by the four livelihood populations in Somalia

**Fig. 3:**
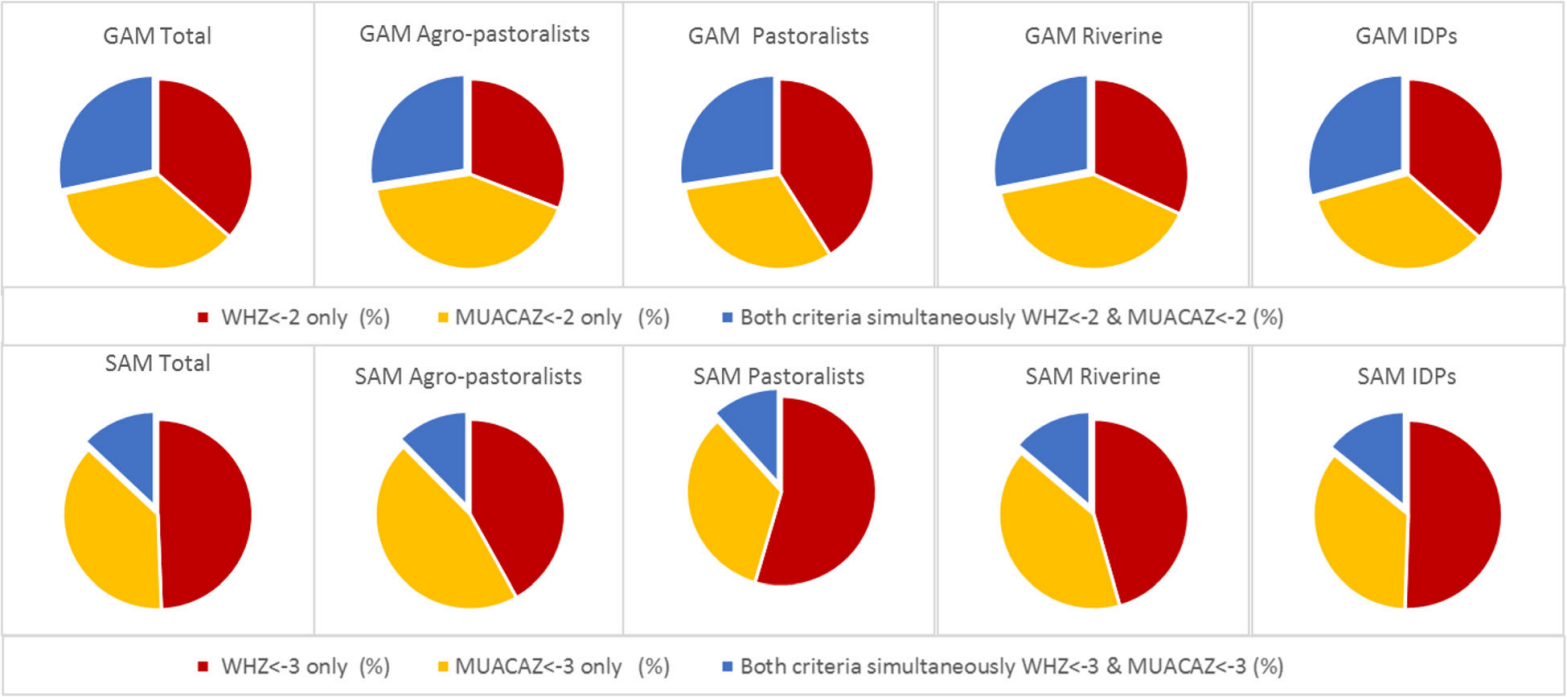
Comparison of acute malnutrition diagnosis by WHZ and/or MUACAZ in four Somalian livelihoods. Proportion of acutely malnourished that are diagnosed as such (GAM first row and SAM second row) either by WHZ alone (red), by MUACAZ alone (yellow) or by both WHZ and MUACAZ simultaneously (blue) for the overall population and by the four livelihood populations in Somalia

At the individual level, the children identified as acutely malnourished by either three of the indicators were not the same. We compared the individual diagnosis between WHZ and the two MUAC measurements alternatively. In Fig. 2 the pie charts represent the proportion of acutely malnourished children diagnosed by either WHZ and/or absolute MUAC and the pie charts in Fig. 3 represent the proportions of acutely malnourished children that were diagnosed as suchby WHZ and/or MUACAZ.

Figures 2 and 3 show that the overlap in the GAM diagnosis by WHZ_− 2_ and MUACAZ-_2_ (children identified as GAM by the two indicators simultaneously) was higher than the overlap between WHZ_− 2_ and MUAC_125_ (28.3 to 18.1%). When the analysis was stratified by livelihood the highest overlap between WHZ_− 2_ and MUAC_125_ was found among agro-pastoralists (20.5%), and the lowest among pastoralists (14.4%) (Fig. 2).

The differences for SAM were more pronounced, and the degree of overlap was significantly lower than for GAM, being higher for children identified by WHZ_− 3_ and MUACAZ_− 3_ (13.0%) than by WHZ_− 3_ and MUAC_115_ (9.6%) for the overall population. The largest discrepancy was found for SAM between WHZ and absolute MUAC in pastoralists children.

Out of the 12,170 children diagnosed as SAM by either WHZ or absolute MUAC, only 2799 children would have been identified if MUAC_115_ was the sole criteria to be used, and 8276 if the children were to be diagnosed by WHZ_− 3_ alone. This difference was more marked among the pastoralists population (see Additional file 1: Table S1).

When comparing the SAM diagnosis by WHZ and by MUACAZ the differences were less pronounced, as out of the 15,052 children diagnosed as SAM by either of these two indicators, 7375 children, would have been diagnosed as severely acutely malnourished if WHZ_− 3_ was the sole indicator used, and 5720 if they were to be diagnosed by MUACAZ_− 3_ alone. The difference again was more pronounced among pastoralists (see Additional file 1: Table S2).

### Factors associated with acute malnutrition

In Table 3 we show the results of the multivariate regression on acute malnutrition (GAM and SAM outcomes) as measured by either three of the indicators.

**Table 3:** Factors associated with the diagnosis of acute malnutrition based on WHZ, MUAC and MUACAZ (*N* = 255,623)

	WHZ						MUAC						MUACAZ					
	WHZ_−2_			WHZ_−3_			MUAC_125_			MUAC_115_			MUACAZ_−2_			MUACAZ_−3_		
	%	*n*	Adj.OR	%	*n*	Adj.OR	%	*n*	Adj.OR	%	*n*	Adj.OR	%	*n*	Adj.OR	%	*n*	Adj.OR
Sex																		
Male	18.2	23,649	Ref.	4.4	5694	Ref.	7.1	9150	Ref.	1.3	1743	Ref.	17.4	22,598	Ref.	3.5	4519	Ref.
Female	13.8	17,391	0.72 (0.71–0.74)	2.9	3704	0.67 (0.64–0.70)	8.6	10,788	1.38 (1.34–1.42)	1.7	2196	1.49 (1.39–1.59)	14.1	17,770	0.82 (0.81–0.84)	2.5	3099	0.76 (0.73–0.80)
Age																		
< 24 months	15.7	13,558	Ref.	4.3	3691	Ref.	16.2	13,951	Ref.	3.6	3081	Ref.	14.7	12,664	Ref.	3.3	2836	Ref.
> =24 months	16.2	27,482	1.04 (1.02–1.07)	3.4	5707	0.78 (0.75–0.82)	3.5	5987	0.19 (0.18–0.20)	0.5	858	0.14 (0.13–0.15)	16.4	27,704	1.19 (1.16–1.22)	2.8	4782	0.91 (0.86–0.95)
Stunting																		
No	15.7	31,993	Ref.	3.5	7110	Ref.	5.8	11,790	Ref.	1.0	2055	Ref.	12.7	25,779	Ref.	2.0	4078	Ref.
Yes	17.3	9047	1.09 (1.06–1.12)	4.4	2288	1.20 (1.15–1.26)	15.6	8148	3.05 (2.96–3.15)	3.6	1884	3.57 (3.35–3.81)	27.8	14,589	2.64 (2.58–2.70)	6.8	3540	3.44 (3.29–3.61)
Total	16.1	41,040		3.7	9398		7.8	19,938		1.5	3939		15.8	40,368		3.0	7618	

Female children were less likely than boys to be diagnosed as acutely malnourished by WHZ or MUACAZ, whereas they were identified as such more than boys (around 40% more) with absolute MUAC. On the other hand, stunted children were identified as acutely malnourished about three times more than non-stunted children when MUACAZ and absolute MUAC were used, compared to 1.3 times, at most, when WHZ was the indicator utilized. Among pastoralists children the impact of stunting on the acute malnutrition outcomes was higher than in the other livelihood groups.

Regarding age, there was a change in the direction of the association when the outcome was GAM or SAM, as older children were more likely to be diagnosed as GAM, and less likely to be diagnosed as SAM when measured by WHZ or by MUACAZ in the overall population. Among agropastoralists and pastoralists populations, the children older than 2 years were also more likely to be diagnosed as SAM when using MUACAZ but not when using WHZ (see Additional file 1: Tables S3 and S4), for model results according to livelihood) . On the contrary, children older than two years were up to 81% and 86% less likely to be diagnosed as GAM or SAM respectively when absolute MUAC was the measure used to diagnose.

## Discussion

We aimed at comparing acute malnutrition estimates as measured by WHZ, absolute MUAC and MUACAZ. A total of 255,623 children aged 6 to 59 months from 19 surveys were included in the analysis. We found an overall GAM prevalence of 16.1% measured by WHZ_− 2_, 7.8% by MUAC_115_ and 15.8% by MUACAZ_− 2_. Similar results were obtained by livelihood system except for the prevalence of GAM in agro-pastoralists and riverine children that was higher using MUACAZ_− 2_ as compared to WHZ_− 2_, and in pastoralists where the prevalence calculated by MUAC_125_ was a third of the prevalence by either WHZ_− 2_ or MUACAZ_− 2_. However, the children identified by either indicator were not the same, being the overlap around 28% between WHZ_− 2_ and MUACAZ_− 2_ diagnosis, and 18% between WHZ_− 2_ and MUAC_125_.

In 1995 the WHO proposed a set of thresholds to classify the severity of malnutrition in emergency situations according to the prevalence of GAM, measured by WHZ_− 2_, that are still widely used (< 5% Acceptable, 5–10% Poor, 10–15% Serious, > 15% Critical) [12]. Up to date there are no thresholds to be used at global level when absolute MUAC is used for nutrition surveillance, and using the same thresholds designed for WHZ is problematic. For the majority of the surveys analysed, the classification of the nutrition situation according to WHO thresholds would have been “Critical” when measured by WHZ, and “Poor” when measured by absolute MUAC.

In response to this limitation, FSNAU developed absolute MUAC emergency thresholds adapted to the Somalia context and based on the calculation of the quintiles of the distribution of absolute MUAC. According to those thresholds, the situation is considered “Critical” if the prevalence of GAM as measured by MUAC_125_ is above 10.7% [2]. However, in eighteen out of the nineteen surveys analysed the situation would nevertheless have been described as “Critical” by WHO thresholds when using WHZ_− 2_ and only “Serious” by FSNAU threshold using MUAC_125_, thus triggering different responses.

According to our results if the GAM is measured by MUACAZ_− 2_ the population estimates are similar to those obtained by WHZ_− 2_, and so they are the resulting classifications according to WHO thresholds in sixteen out of the nineteen surveys analysed.

One of the main arguments for not using MUACAZ it is the age dependency of the indicator, as it is reported that in certain contexts with a high burden of acute malnutrition, age is difficult to ascertain accurately [9]. This may not be the case in Somalia as the field teams have extensive experience in collecting this information due to the high frequency and long history of surveys being conducted in the country. In the sample analyzed the proportion of children 6–23 months old compared to the 24–59 months age group followed the 1:2 expected proportion ratio. Moreover, in other emergency nutrition surveillance systems data is collected on both weight-for-age and absolute MUAC, suggesting that the age collection is feasible to collect with accuracy [3]. There is also evidence from Bangladesh showing that MUAC-for-age provides comparable information to weight-for-height in the context of nutritional surveillance of populations, and especially for assessing seasonal changes [15].

We recommend to revise the age dependency limitation in view of the importance of bringing age in the estimation of acute malnutrition when using the MUAC measurement. As other authors have already suggested, it could also be possible to construct tally sheets for MUAC divided by age groups in order to minimise the constraints regarding age ascertainment and its accuracy [17].

In relation to the individual screening of acutely malnourished children the results obtained by either indicator did not converge. The discrepancy observed between absolute MUAC and by WHZ in children screening had already been studied [17, 18] and taken into account in WHO-UNICEF guidelines [19, 20], but seldom is published on the comparison of children diagnosed as acutely malnourished according to MUACAZ or to WHZ.

The results obtained by Grellety and Golden in their recent review of 47 countries [7] are consistent with our findings regarding the WHZ /MUAC discrepancy in Somalia overall, and the differences we found according to livelihood system were similar to those found by these authors in Ethiopia. Previous studies conducted in Ethiopia found similar results and argued that the prevalence of acute malnutrition by WHZ was overestimated due to differences in body shape such as relatively longer legs that contribute to the total height [21]. However, recent studies refute that this factor alone can explain the discrepancies observed, concluding that they may be due to the combination of different anthropometric characteristics that are expressed differently across populations [8, 11].

Moreover, our results disagree with the hypothesis of pastoralist populations showing systematic higher estimates for acute malnutrition as compared to other populations in the same context [22] as they show that the GAM prevalence (according to any of the three measurements) among the pastoralist children was lower than in any of the other livelihood populations studied.

Finally, our results show that age, sex and stunting status of the children also play a role in their likelihood of being diagnosed as acutely malnourished depending on the indicator to be used. Female, younger, and stunted children were more likely to be identified as acutely malnourished if the indicator to be used was absolute MUAC, whereas boys older than two years and stunted were more likely to be diagnosed as GAM by WHZ. Our results confirm those of other studies conducted in countries as Philippines, Chad, South Sudan and Bangladesh [8], Kenya [23] or South Sudan [7], and some authors have offered the explanation that MUAC is mainly a measure of muscle mass, and that muscle mass is reduced in stunted children [24, 25]. Interestingly, we found that older boys were more likely to be identified as experiencing GAM by MUACAZ (similar to WHZ results), but also that MUACAZ was the indicator to be more strongly associated with stunting. Consistently, a study in Kenya reported that upper arm muscle area Z-score, an age and gender adjusted measure of absolute lean body mass, explained most of the variability in the progression of stunting among school-aged children over a two year-period [26], suggesting that age adjusted measurements may be more appropriate in the interpretation of weight-for-height and MUAC measurements discrepancies in relation to stunting.

### Strengths and limitations

The sample characteristics in terms of size and number of surveys allowed for a high precision in the analysis, and for the stratification by livelihood system. The so called IDPs livelihood was over-represented in the overall population as recent data indicated that the proportion of IDPs in Somalia population was around 14% [27], thus the importance of the stratified analysis.

The data were collected in field conditions which may have an impact on the accuracy of measurements, although the FSNAU survey enumerators long time experience and routinely training may have minimized this limitation.

Another limitation of our study is that the analysis was performed on a single country and results cannot be generalizable for other countries.

## Conclusions

The estimates of acute malnutrition by MUACAZ are convergent with the estimations reported by WHZ, as opposed to the high discrepancies found in relation to the absolute MUAC estimates, in our study population. We recommend further research on the MUACAZ indicator in the estimations of GAM in other contexts in which WHZ cannot be collected, as well as the reporting of this indicator in nutrition surveillance reports in Somalia whenever possible.

We encourage the inclusion of MUACAZ in the array of outcomes to be calculated by the available software for nutrition surveillance estimates like the Emergency Nutrition Assessment (ENA) software.

In relation to screening for program admission, we recommend adherence to the current guidelines of using both absolute MUAC and weight-for-height for the screening of severely and moderately malnourished children, and promote research on the role that MUACAZ or alternative indicators can play in this type of surveillance.

## Additional file

Additional file 1: **Table S1.** Comparison of acute malnutrition diagnosis by WHZ and/or absolute MUAC in four Somalian livelihoods. Proportion of children with acute malnutrition (GAM or SAM) as defined by WHZ and/or absolute MUAC (see Methods section for definitions) that are diagnosed as malnourished by WHZ only, by absolute MUAC only, or by both criteria simultaneously. **Table S2.** Comparison of acute malnutrition diagnosis by WHZ and/or MUACAZ in four Somalian livelihoods. Proportion of children with acute malnutrition (GAM or SAM) as defined by WHZ and/or absolute MUACAZ (see Methods section for definitions) that are diagnosed as malnourished by WHZ only, by MUACAZ only, or by both criteria simultaneously. **Table S3.** Factors associated with GAM diagnosis based on WHZ, absolute MUAC and MUACAZ by livelihood. Results of the multivariate regression on GAM as measured by either three of the indicators (WHZ, MUAC and MUACAZ) conducted in each of the four livelihood populations. **Table S4.** Factors associated with SAM diagnosis bases on WHZ, MUAC and MUACAZ by livelihood. Results of the multivariate regression on SAM as measured by either three of the indicators (WHZ, MUAC and MUACAZ) conducted in each of the four livelihood populations. (PDF 211 kb)

## Abbreviations

ENA: Emergency Nutrition Assessment
FSNAU: Food Security and Nutrition Analysis Unit
GAM: Global Acute Malnutrition
IDPs: Internally Displaced Persons
MUAC: Mid-Upper Arm Circumference
MUACAZ: Mid-Upper Arm Circumference Z-score)
SAM: Severe Acute Malnutrition
SMART: Standardized Monitoring and Assessment of Relief and Transitions
UNICEF: United Nations International Children’s
WHO: World Health Organization
WHZ: Weight-for-Height Z-score
