# Supplementary material for: MUAC-for-age more useful than absolute MUAC for nutritional surveillance in Somalia: results from nineteen cross-sectional surveys (2007–2016)
Source: BMC Nutr. 2018 Feb 21;4:8. doi: 10.1186/s40795-018-0213-3 (PMC7050741; doi:10.1186/s40795-018-0213-3)
Supplement: Supplementary file 1 — Table S1. Comparison of acute malnutrition diagnosis by WHZ and/or absolute MUAC in four Somalian livelihoods. Proportion of children with acute malnutrition (GAM or SAM) as defined by WHZ and/or absolute MUAC (see Methods section for definitions) that are diagnosed as malnourished by WHZ only, by absolute MUAC only, or by both criteria simultaneously. Table S2. Comparison of acute malnutrition diagnosis by WHZ and/or MUACAZ in four Somalian livelihoods. Proportion of children with acute malnutrition (GAM or SAM) as defined by WHZ and/or absolute MUACAZ (see Methods section for definitions) that are diagnosed as malnourished by WHZ only, by MUACAZ only, or by both criteria simultaneously. Table S3. Factors associated with GAM diagnosis based on WHZ, absolute MUAC and MUACAZ by livelihood. Results of the multivariate regression on GAM as measured by either three of the indicators (WHZ, MUAC and MUACAZ) conducted in each of the four livelihood populations. Table S4. Factors associated with SAM diagnosis bases on WHZ, MUAC and MUACAZ by livelihood. Results of the multivariate regression on SAM as measured by either three of the indicators (WHZ, MUAC and MUACAZ) conducted in each of the four livelihood populations. (PDF 211 kb) [file 40795_2018_213_MOESM1_ESM.pdf]

Additional File 1

Supplementary table 1: Comparison of acute malnutrition diagnosis by WHZ and/or absolute MUAC in four Somalian livelihoods

		Livelihood			
	**Total**	**Agro-pastoralist**	**Pastoralist**	**Riverine**	**IDPs**
**Indicators for GAM**					
WHZ_{-2} or/and $MUAC_{125}$	51,619	9,064	16,597	5,567	20,391
WHZ_{-2} only	61.4	55.3	69.4	57.4	58.7
$MUAC_{125}$ only (%)	20.5	24.2	16.3	23.5	21.5
WHZ_{-2} & $MUAC_{125}$ (%)	18.1	20.5	14.4	19.1	19.9
**Indicators for SAM**					
WHZ_{-3} or/and $MUAC_{115}$	12,170	2,184	3,553	1,355	5,078
WHZ_{-3} only (%)	67.6	66.2	75	65.8	63.6
$MUAC_{115}$ only (%)	22.8	23.9	17.9	24.3	25.3
WHZ_{-3} and $MUAC_{115}$ (%)	9.6	9.9	7.1	9.9	11.1

Proportion of children with acute malnutrition (GAM or SAM) as defined by WHZ and/or absolute MUAC (see Methods section for definitions) that are diagnosed as malnourished by WHZ only , by absolute MUAC only, or by both criteria simultaneously.

Supplementary table 2: Comparison of acute malnutrition diagnosis by WHZ and/or MUACAZ in four Somalian livelihoods

		Livelihood			
	**Total**	**Agro-pastoralist**	**Pastoralist**	**Riverine**	**IDPs**
**Indicators for GAM**					
WHZ_{-2} or/and $MUACAZ_{-2}$	63,478	11,757	20,319	7,105	24,297
WHZ_{-2} only (%)	36.4	30.9	41	31.9	36.5
$MUACAZ_{-2}$ only (%)	35.4	41.6	31.6	40.1	34.1
WHZ_{-2} & $MUACAZ_{-2}$ (%)	28.3	27.5	27.4	28.1	29.4
**Indicators for SAM**					
WHZ_{-3} or/and $MUACAZ_{-3}$	15,052	3,060	4,403	1,729	5,860
WHZ_{-3} only (%)	49.4	41.9	54.5	45.6	50.6
$MUACAZ_{-3}$ only (%)	37.6	45.7	33.8	40.7	35.3
WHZ_{-3} and $MUACAZ_{-3}$ (%)	13.0	12.4	11.7	13.8	14.2

Proportion of children with acute malnutrition (GAM or SAM) as defined by WHZ and/or absolute MUACAZ (see Methods section for definitions) that are diagnosed as malnourished by WHZ only , by MUACAZ only, or by both criteria simultaneously.

Supplementary table 3 Factors associated with GAM diagnosis based on WHZ, absolute MUAC and MUACAZ by livelihood

S3-1 Factors associated with the diagnosis of GAM among agro-pastoralists (n=42 132)

	WHZ-2			MUAC125			MUACAZ-2		
	%	n	Adj.OR	%	n	Adj.OR	%	n	Adj.OR
Sex									
Male	18.7	3 987	Ref.	9.2	1 966	Ref.	21.5	4 583	Ref.
Female	13.9	2 882	0.70 (0.67-0.74)	10.0	2 089	1.21 (1.13-1.29)	17.0	3 540	0.80 (0.76-0.84)
Age									
<24 months	15.6	2 215	Ref.	17.5	2 481	Ref.	16.3	2 314	Ref.
>=24 months	16.7	4 654	1.08 (1.02-1.14)	5.6	1 574	0.27(0.25-0.29)	20.8	5 809	1.34 (1.27-1.41)
Stunting									
No	16.0	4 975	Ref.	7.4	2 289	Ref.	15.1	4 700	Ref.
Yes	17.1	1 894	1.05 (0.99-1.11)	16.0	1 766	2.62 (2.45-2.81)	31.0	3 423	2.46 (2.34-2.59)
Total	16.3	6 869		9.6	4 055		19.3	8,123	

S3-2 Factors associated with the diagnosis of GAM in pastoralists (n=89 876)

	WHZ-2			MUAC125			MUACAZ-2		
	%	n	Adj.OR	%	n	Adj.OR	%	n	Adj.OR
Sex									
Male	17.5	8 079	Ref.	5.1	2 353	Ref.	14.6	6 774	Ref.
Female	13.4	5 820	0.74 (0.71-0.76)	6.3	2 729	1.36 (1.28-1.44)	12.0	5 206	0.83 (0.80-0.87)
Age									
<24 months	12.4	3 670	Ref.	11.3	3 332	Ref.	10.1	2 968	Ref.
>=24 months	17.0	10 229	1.44 (1.38-1.50)	2.9	1 750	0.23 (0.22-0.25)	14.9	9 012	1.61 (1.54-1.68)
Stunting									
No	15.2	11 958	Ref.	4.7	3 688	Ref.	11.6	9 132	Ref.
Yes	17.2	1 941	1.13 (1.07-1.19)	12.3	1 394	2.98 (2.78-3.19)	25.2	2 848	2.56 (2.44-2.69)
Total	15.5	13 899		5.7	5 082		13.3	11 980	

S3-3 Factors associated with the diagnosis of GAM in riverine (n=26 603)

	WHZ-2			MUAC125			MUACAZ-2		
	%	n	Adj.OR	%	n	Adj.OR	%	n	Adj.OR
Sex									
Male	18.0	2 435	Ref.	8.1	1 103	Ref.	19.9	2 695	Ref.
Female	14.0	1 823	0.74 5(0.69-0.79)	9.7	1 271	1.39 (1.27-1.52)	16.5	2 146	0.84 (0.79-0.90)
Age									
<24 months	16.7	1 479	Ref.	18.1	1 600	Ref.	16.3	1 447	Ref.
>=24 months	15.7	2 779	0.92 (0.86-0.99)	4.4	774	0.21 (0.19-0.23)	19.1	3 394	1.27 (1.18-1.36)
Stunting									
No	16.3	3 183	Ref.	6.9	1 338	Ref.	14.8	2 877	Ref.
Yes	15.1	1 075	0.88 (0.82-0.95)	14.5	1 036	2.33 (2.13-2.55)	27.6	1 964	2.19 (2.05-2.34)
Total	16.0	4 258		8.9	2 374		18.2	4 841	

S3-4 Factors associated with the diagnosis of GAM in IDPs (n=97 012)

	WHZ-2			MUAC125			MUACAZ-2		

	%	n	Adj.OR	%	n	Adj.OR	%	n	Adj.OR
Sex									
Male	18.9	9 148	Ref.	7.7	3 728	Ref.	17.6	8 546	Ref.
Female	14.2	6 866	0.71 (0.69-0.74)	9.7	4 699	1.46 (1.39-1.53)	14.2	6 878	0.82 (0.79-0.85)
Age									
<24 months	16.4	6 194	Ref.	19.4	6 538	Ref.	17.6	5 935	Ref.
>=24 months	15.5	9 820	0.82 (0.79-0.85)	3.0	1 889	0.13 (0.13-0.14)	15.0	9 489	0.89 (0.86-0.93)
Stunting									
No	16.0	11 877	Ref.	6.0	4 475	Ref.	12.2	9 070	Ref.
Yes	18.1	4 137	1.11 (1.06-1.15)	17.2	3 952	3.12 (2.97-3.27)	27.7	6 354	2.69 (2.59-2.79)
Total	16.5	16,014		8.7	8 427		15.9	15 424	

Supplementary table 4 Factors associated with SAM diagnosis based on WHZ, MUAC and MUACAZ by livelihood

S4-1 Factors associated with the diagnosis of SAM among agro-pastoralists (n=42,132)

	WHZ$_{-3}$			MUAC$_{115}$			MUACAZ$_{-3}$		
	%	n	Adj.OR	%	n	Adj.OR	%	n	Adj.OR
Sex									
Male	4.7	996	Ref.	1.6	331	Ref.	5.0	1 055	Ref.
Female	3.2	667	0.68 (0.62-0.76)	2.0	407	0.42 (1.22-1.65)	3.5	721	0.75 (0.68-0.82)
Age									
<24 months	4.3	602	Ref.	3.7	520	Ref.	3.5	499	Ref.
>=24 months	3.8	1 061	0.89 (0.80-0.98)	0.8	218	0.20 (0.17-0.23)	4.6	1,277	1.29 (1.16-1.44)
Stunting									
No	3.7	1 161	Ref.	1.3	390	Ref.	2.9	892	Ref.
Yes	4.5	502	1.19 (1.07-1.32)	3.2	348	2.81 (2.42-3.26)	8.0	884	2.86 (2.60-3.15)
Total	4.0	1 663		1.8	738		4.2	1 776	

S42 Factors associated with the diagnosis of SAM in pastoralists (n=89 876)

	WHZ$_{-3}$			MUAC$_{115}$			MUACAZ$_{-3}$		
	%	n	Adj.OR	%	n	Adj.OR	%	n	Adj.OR
Sex									
Male	3.9	1 810	Ref.	0.9	413	Ref.	2.6	1 211	Ref.
Female	2.5	1 106	0.65 (0.60-0.70)	1.1	475	1.40 (1.22-1.60)	1.8	793	0.75 (0.69-0.82)
Age									
<24 months	3.1	928	Ref.	2.1	621	Ref.	1.9	572	Ref.
>=24 months	3.3	1 988	1.05 (0.97-1.14)	0.4	267	0.21 (0.18-0.24)	2.4	1 432	1.27 (1.15-1.40)
Stunting									
No	3.1	2 468	Ref.	0.7	559	Ref.	1.7	1 316	Ref.
Yes	4.0	448	1.22 (1.10-1.35)	2.9	329	4.25 (3.70-4.89)	6.1	688	3.73 (3.39-4.10)
Total	3.2	2 916		1.0	888		2.2	2 004	

S4-3 Factors associated with the diagnosis of SAM in riverine (n=26 603)

	WHZ$_{-3}$			MUAC$_{115}$			MUACAZ$_{-3}$		
	%	n	Adj.OR	%	n	Adj.OR	%	n	Adj.OR

Sex									
Male	4.6	622	Ref.	1.6	216	Ref.	4.1	555	Ref.
Female	3.1	404	0.67 (0.59-0.76)	1.9	247	1.39 (1.15-1.67)	3.0	386	0.78 (0.68-0.89)
Age									
<24 months	4.6	403	Ref.	4.1	362	Ref.	3.9	348	Ref.
>=24 months	3.5	623	0.77 (0.67-0.87)	0.6	101	0 .14 (0.11-0.17)	3.3	593	0.89 (0.78-1.02)
Stunting									
No	3.8	743	Ref.	1.2	237	Ref.	2.5	486	Ref.
Yes	4.0	283	0.99 (0.86-1.14)	3.2	226	2.58 (2.14-3.11)	6.4	455	2.59 (2.27-2.95)
Total	3.9	1 026		1.7	463		3.5	941	

S4-4 Factors associated with the diagnosis of SAM in IDPs (n=97 012)

	Weight-for-height			$MUAC_{115}$			$MUACAZ_{-3}$		
	%	n	Adj.OR	%	n	Adj.OR	%	n	Adj.OR
Sex									
Male	4.7	2 266	Ref.	1.6	783	Ref.	3.5	1 698	Ref.
Female	3.2	1 527	0.67 (0.63-0.71)	2.2	1 067	1.55 (1.41-1.71)	2.5	1 199	0.76 (0.71-0.82)
Age									
<24 months	5.2	1 758	Ref.	4.7	1 578	Ref.	4.2	1 417	Ref.
>=24 months	3.2	2 035	0.61 (0.57-0.65)	0.4	272	0.10 (0.08-0.11)	2.3	1 480	0.61 (0.56-0.65)
Stunting									
No	3.7	2 738	Ref.	1.2	869	Ref.	1.9	1 384	Ref.
Yes	4.6	1 055	1.16 (1.08-1.25)	4.3	981	3.37 (3.07-3.71)	6.6	1 513	3.48 (3.23-3.75)
Total	3.9	3 793		1.9	1 850		3.0	2 897	
